# Augmentative transcranial magnetic stimulation over the left orbitofrontal cortex in patients with treatment-resistant obsessive-compulsive disorder: An acute and follow-up study

**DOI:** 10.1016/j.nsa.2025.105511

**Published:** 2025-02-14

**Authors:** Matteo Vismara, Sara Torriero, Kevin La Monica, Beatrice Benatti, Luca Larini, Chiara Bucca, Nicolaja Girone, Monica Bosi, Bernardo Dell’Osso

**Affiliations:** a University of Milan, Department of Mental Health, Department of Biomedical and Clinical Sciences Luigi Sacco, Milan, Italy; b “Aldo Ravelli” Center for Neurotechnology and Brain Therapeutic, University of Milan, Milan, Italy; c Department of Psychiatry and Behavioral Sciences, Bipolar Disorders Clinic, Stanford University, CA, USA

**Keywords:** Obsessive-compulsive disorder, Transcranial magnetic stimulation, Treatment resistance, Orbitofrontal cortex

## Abstract

**Background:**

Obsessive-compulsive disorder (OCD) is a disabling and chronic medical condition which impairs the overall functioning and the quality of life of affected individuals. At the current moment up to 60% of patients do not show a satisfactory response, and among alternative approaches for treatment-resistant OCD, repetitive transcranial magnetic stimulation (rTMS) showed promising results in terms of efficacy and tolerability. Despite this, stimulation parameters are still heterogeneous, and additional investigations are needed to support these data.

**Methods:**

OCD patients with characteristic of treatment resistance were included in this open-label trial. The stimulation protocol consisted of one daily session, five days a week for three weeks, for a total of 15 sessions. The left orbitofrontal cortex (OFC) was the target, stimulated at 80% of the motor threshold, with a frequency of 1 Hz, 600 pulses per session. All patients maintained fixed medication doses during the trial. Primary outcome measures comprised the Yale-Brown Obsessive-Compulsive Scale (Y-BOCS), the Hamilton Anxiety Rating Scale (HAM-A), the Hamilton Depression Rating Scale (HAM-D), the Sheehan Disability Scale (SDS), and the Clinical Global Impression Scale (CGI-S) scores, assessed at baseline (T0), at the end of the treatment (T1), and after a follow-up of one month (T2). We identified responders with a Y-BOCS reduction of ≥35% at T1. General linear model repeated measures were used to compare scores at psychometric scales, chi-squared test was used to compare variables between responders and non-responders.

**Results:**

Thirteen patients completed the psychometric assessment (males: 69.2%; females: 30.8%, mean age: 43.1 ± 10.2 years). We observed a significant reduction at the end of the treatment on the Y-BOCS (T0:23.4 ± 8.9 - T1:18.2 ± 7.2, p = 0.009), HAM-A (T0:13.8 ± 7.4-T1:8.6 ± 4, p = 0.006), HAM-D (T0:13.2 ± 5.8 - T1:9.5 ± 3.6, p = 0.014), SDS (T0:22.7 ± 6.2 - T1:18.3 ± 5.1, p = 0.008), and CGI-S scores (T0:4.8 ± 0.8 - T1:4.3 ± 0.9, p = 0.027). Among all timepoints, a trend of significance for reduction of Y-BOCS and HAM-A scores emerged (p = 0.075 and p = 0.077, respectively), while HAM-D scores were significantly reduced after one month (p = 0.047). Responders constituted 30.8% (N = 4) of the sample. Worse clinical variables were more frequently observed in non-responders compared to responders: a higher rate of psychiatric familiarity and a higher rate of lifetime suicidal ideation. The only side effect reported was mild and transient headache during stimulation (N = 1).

**Conclusions:**

Our data support the efficacy and tolerability of rTMS over the left OFC on obsessive-compulsive, depressive, and anxious symptoms in treatment-resistant OCD, overall associated with a reduction of disability and functional impairment. Additionally, one third of patients showed a response and results suggest a maintenance of efficacy after one month follow-up.

## Highlights

• rTMS applied at 1 Hz to the OFC showed to ameliorate obsessive-compulsive symptoms in patients with treatment resistant OCD.
• At the end of the treatment, also anxious and depressive symptoms decreased, and patients showed an overall reduction of disability and functional impairment.
• The procedure was well tolerated, headache was the only side effect reported.

## 1 Introduction

Obsessive-compulsive disorder (OCD) is a highly disabling psychiatric condition, which affects nearly 2–3% of the worldwide population (Carmi et al., 2022). Several evidence-based psychological and pharmacological treatments have been approved for OCD, however, as high as 40–60% of patients do not respond satisfactory to standard treatment options (Fineberg et al., 2020). Patients showing characteristics of partial/non-response are associated with a worse clinical picture, including higher risk of suicide and a higher illness-related disability for patients and their caregivers (Pallanti et al., 2002).

Among alternative approaches for treatment-resistant OCD (TR-OCD), neuromodulation techniques have been proposed, aimed to restore specific neural circuits that are affected in OCD (i.e., the cortico-striatal-thalamic circuit) (Fettes et al., 2017). These include surgical procedures like capsulotomy, cingulotomy, and deep brain stimulation, or non-surgical ones like repetitive transcranial magnetic stimulation (rTMS) or transcranial direct current stimulation (D.J. et al., 2022). rTMS is potentially the most preferable approach, considering it is non-invasive compared to other techniques and randomised-controlled trials and meta-analytic data confirming its efficacy and tolerability (Liang et al., 2021), (Steuber and McGuire, 2023). In this light, rTMS, in the form of deep-TMS, has received in 2019 the FDA approval for the treatment of patients with TR-OCD (Carmi et al., 2019). In the only published investigation that compared augmentative rTMS to augmentative antipsychotic drugs in 50 patients with OCD refractory to selective serotonin reuptake inhibitors (SSRI), 68% of patients were responders in the rTMS group compared to only 24% in the antipsychotic group (Pallanti et al., 2016). Several randomized-controlled trials have been published on the use of rTMS in patients with TR-OCD, however these results are inconsistent, probably related to the wide variety of stimulation protocols used (e.g., target (which specific brain region or cortical vs. deeper stimulation), laterality, frequency (low vs. high frequency), duration, and length of treatment). Different brain regions have been targeted, including the orbitofrontal cortex (OFC), the dorsolateral prefrontal cortex (DLPFC), the anterior cingulate cortex (ACC), the supplementary motor area (SMA), and the medial prefrontal cortex (Tyagi et al., 2019). Up to date, the DLPFC is the most investigated brain area, with some robust data confirming that targeting this area led to an improvement of obsessive and compulsive symptoms. However, also the OFC is potentially an ideal target, warranting further investigations. Indeed, the OFC is a cortical node within neural circuitry specifically implicated in improvement in OCD, alongside reward processing and antidepressant effects (Berlim et al., 2013). However, meta-analytic data showed controversial results, as earlier meta-analyses identified the OFC as the most efficacious target (Berlim et al., 2013), but later meta-analyses favored the SMA (Zhou et al., 2017), (Rehn et al., 2018) or the bilateral DLPFC as the most effective target in OCD (Liang et al., 2021), (Perera et al., 2021), (Fitzsimmons et al., 2022).

**Fig. 1:**
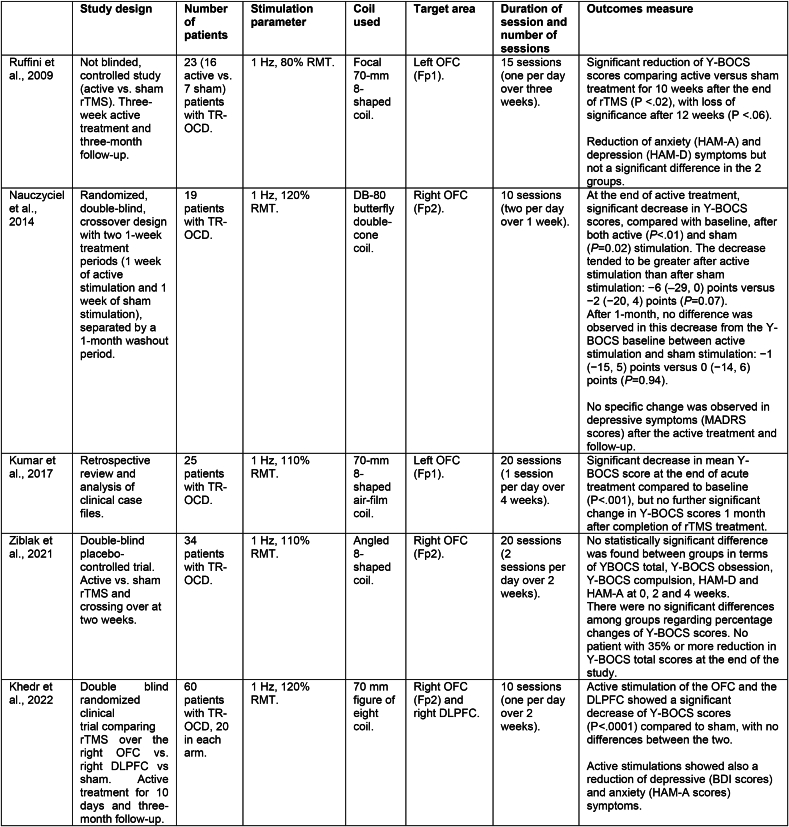
Comparison of literature studies investigating rTMS over the orbitofrontal cortex. *Legend:* BDI: Beck Depression Inventory Scale; DLPFC: the dorsolateral prefrontal cortex; HAM-A: Hamilton Anxiety Rating Scale; HAM-D: Hamilton Rating Scale for Depression; MADRS: Montgomery and Asberg Depression Rating Scale; RMT: resting motor threshold; TR-OCD: treatment resistant OCD; Y-BOCS: Yale-Brown obsessive-compulsive scale.

To the best of our knowledge, there are only limited studies that primarily investigated rTMS applied to the OFC in patients with OCD (Ruffini et al., 2009), (Nauczyciel et al., 2014), (Khedr et al., 2022), (Kumar et al., 2018), (Ziblak et al., 2021). Fig. 1 summarizes literature studies on this topic.

The first randomized placebo-controlled study (Ruffini et al., 2009) included 23 patients with medication-resistant OCD in two arms (16 received active rTMS (1 Hz) and 7 received sham rTMS on the left OFC). Active vs. sham treatment showed a significant reduction on the Y-BOCS total scores at the end of the stimulation that was maintained for 10 weeks, with loss of significance after 12 weeks. Moreover, both groups showed a reduction of anxiety and depression symptoms. In another randomized placebo-controlled study (Nauczyciel et al., 2014), 19 medication-resistant patients with OCD were divided into two groups (active 1 Hz rTMS vs. sham, two sessions per day for one week on the left OFC). After a wait period of one month for the therapeutic effects to disappear, the groups crossed over to the other treatment condition. OFC metabolism was studied using a positron emission tomography (PET). After one week of treatment, both groups showed a significant decrease in Y-BOCS scores, and between-group differences showed a trend towards statistical significance. Active vs. sham PET scan contrasts showed that stimulation was related to a bilateral decrease in the metabolism of the OFC. The authors concluded that the OFC region could be a valid target for the treatment of OCD (Nauczyciel et al., 2014). A further retrospective review and analysis of clinical case files of 25 patients with medication-refractory OCD who had received 20 sessions of rTMS (1 Hz) over the left OFC showed that the procedure led to a significant reduction in clinical symptoms, with 52% of patients meeting criteria for partial response and 44% of complete response (Kumar et al., 2018).

Controversial results on the efficacy of augmentative rTMS targeting the right-OFC emerged in a recent randomized placebo-controlled study (Ziblak et al., 2021). In detail, 34 medication-resistant OCD patients were divided into two groups (active 1Hz vs. sham rTMS) in a crossover design. No statistically significant difference was found between groups in terms of Y-BOCS scores at the end of the treatment. Also, among all OCD patients participating in the study, there was no patient with 35% or more reduction in Y-BOCS total scores at the end of the study, overall suggesting that rTMS applied at 1 Hz targeting the right OFC is not effective for medication-resistant OCD (Ziblak et al., 2021). The opposite result, compared to the three previous studies, could be related to the different area targeted (left vs. right OFC). However, a recent randomized-controlled trial showed a positive effect of this area. In this study, 60 patients with OCD were randomly allocated to three treatment groups (1Hz rTMS to the left DLPFC vs. the right OFC vs. and sham, 20 patients in each arm, 10 sessions) and both active treatments were superior to placebo, with a significantly higher percentage of responders in both active groups (DLPFC: 60%, OFC: 65%) compared to the sham group (0%). No significant difference between the two active protocols emerged (Khedr et al., 2022).

Considering this background, additional studies investigating the efficacy of rTMS, in particular over the OFC, should be encouraged. In the present open label, not controlled, exploratory study, we aimed to measure the efficacy of 1 Hz rTMS over the left OFC in a sample of patients with TR-OCD. We hypothesized that low-frequency stimulation of the OFC would lead to a reduction in obsessive-compulsive symptoms, as measured on the Y-BOCS. Moreover, we aimed to measure changes in other symptoms related to OCD like depression and anxiety. Lastly, data on safety and tolerability of this procedure will be measured. Although this is not a randomized double-blind clinical trial, we expected to find data of clinical significance that would help to increase the generalizability of the treatment potential of rTMS in OCD and that may contribute to untangle the optimal treatment protocol, in terms of optimal parameters of the stimulation.

## 2 Material and methods

### 2.1 Study sample

**Fig. 2:**
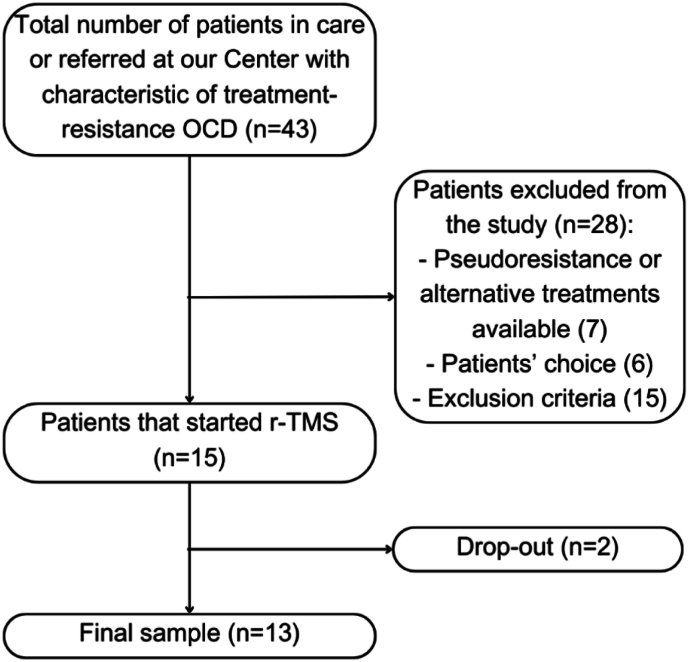
Flowchart of patients' recruitment.

This interventional, open-label, study was conducted at “Luigi Sacco” University Hospital in Milan, Italy. Patients were recruited from a tertiary psychiatric service dedicated to the diagnosis and treatment of outpatients with OCD, where patients were in care or referred from other psychiatric centers. Patients' recruitment started in January 2021 and was still ongoing at the time of the statistical analysis (February 2024). Inclusion criteria were adult subjects (over age 18 years) with a diagnosis of OCD confirmed by trained psychiatrists through the administration of the Structured Clinical Interviews for DSM-5 (SCID), clinical version (First et al., 2016). In the case of psychiatric comorbidities, OCD had to be considered the primary disorder and directly responsible for obsessive-compulsive symptoms. Patients have to show characteristic of treatment resistance, that was defined as one or two failed trials of adequately dosed SSRI that have lasted at least 8–12 weeks (Pallanti et al., 2002). At the clinical assessment, the rTMS protocol was explained to patients which voluntarily choose if participating in the trial. Lastly, psychopharmacological treatment had to be maintained unchanged for the 4 weeks preceding rTMS and during the trial. Exclusion criteria involved the presence of neurological disorders (epilepsy or familiarity for epilepsy, previous significant head trauma, brain surgery, loss of consciousness for minimum 15 min), pregnancy or lactation, significant medical and/or psychiatric comorbidities, substance abuse in the last 3 months, pacemakers or electrical stimulation devices, metallic clips, severe cardiac disorders, hypertension, and sleep apnea. Patients might not be included in the trial in case of pseudo-resistance (e.g., OC symptoms not primarily linked to OCD), if there were alternative approaches for TR-OCD as per clinical judgment or patients' preference. Fig. 2 represents patients’ recruitment.

The following socio-demographic and clinical characteristics were collected: age, age at onset, gender, family history, ethnicity, marital status, employment, educational status, living standards, psychiatric and medical comorbidities, stressors at OCD onset, duration of illness, duration of untreated illness (DUI), previous and current pharmacological and psychological treatments, lifetime and current suicidal ideation and attempts, number of suicidal attempts, and number of hospitalizations.

The study was approved by the local Ethics Committee (number 721/2020, "Comitato Etico Milano Area 1), and participants were treated in accordance with the Declaration of Helsinki (World Medical Association Declaration of, 2013). All participants gave written informed consent before the study procedure to use their anonymized data for research purposes.

### 2.2 rTMS protocol

Stimulation was administered using a MagVenture Magpro R30 high-performance TMS stimulator connected to an MCF-B70 figure-of-eight coil (MagVenture Company, Farum, DK). Participants were scheduled to be administered a total of 15 sessions (5 sessions per week for 3 consecutive weeks). The identification of the resting motor threshold (RMT) and of the target area was performed according to standard procedures (Rossini et al., 2015). RMT was considered as the minimum TMS intensity producing a motor evoked potential in the contralateral abductor pollicis brevis in at least 50% of trials (Pascual-Leone et al., 1992). The target area was identified following the current available literature (Tyagi et al., 2019), using the International 10–20 EEG system (identified with the Fp1 location), with stimulation parameters chosen within those recommended by recent International Safety Guidelines (Sharbrough et al., 1991): left OFC, 1 Hz frequency, intensity of stimulus 80% of RMT, 600 stimuli per session. No progressive titration of the intensity of the stimulation was required.

### 2.3 Outcome measures

To measure the efficacy of rTMS, patients were administered a set of psychometric scales at baseline (T0) and at the end of the treatment (T1). Moreover, patients were assessed at a follow-up time of one month (T2), to investigate changes in the symptomatology over time, after the end of the active stimulation.

The primary outcome measure was changes in the obsessive-compulsive symptoms, as assessed with the Y-BOCS (Goodman et al., 1989), a widely used scale to measure the severity of OCD. The severity of obsessions and compulsions are graded on a scale ranging from 0 to 4 points, with a maximum score of 20 in obsessions and compulsions, respectively.

To measure a possible effect of the procedure on other symptoms, the following psychometric scales were also administered: the Hamilton Rating Scale for Depression (HAM-D, 21 items) (Hamilton, 1960) to measure depressive symptoms, and the Hamilton Anxiety Rating Scale (HAM-A) (Hamilton, 1959) to measure anxious symptoms. To have a global valuation of the severity of the disease, the Clinical Global Impression-Severity Scale (CGI) was also used (Guy, 1976). Additionally, to have a subjective evaluation of the impact of the disease on daily life, the Sheehan Disability Scale (SDS) was included (Sheehan et al., 1996).

Raters were clinicians with a certified expertise in the psychometric assessment and were distinct from clinicians providing the treatment. For every patient, the same rater performed the assessment at each time-point.

All questionnaires were administered at baseline, at T1, and T2. To categorize patients with response to treatment, the sample was divided into two subgroups: “responders” were defined as those patients who showed a Y-BOCS score reduction ≥35% at T1, alternatively as “non-responders”.

### 2.4 Safety and tolerability measures

To assess safety and tolerability of the procedure, patients were daily monitored before and during each rTMS session, collecting spontaneously reported side effects and adverse events. Clinical judgment was used to investigate if these events were related to the stimulation itself and, if so, registered accordingly. Rates and reasons for early termination were also registered.

### 2.5 Statistical analyses

Primary analyses were performed comparing scores on clinical scales at T0 and T1, to evaluate the short-term effects of the treatment. Secondary analyses were extended to T2 to explore delayed effects, and LSD pairwise comparisons were performed when required.

General linear model repeated measures were used to compare scores at psychometric scales, with time as within factor (2 levels for primary analyses: T0 vs. T1; 3 levels for secondary analyses: T0 vs. T1, T2). At one-month follow-up, two patients were missing, therefore the secondary analyses were performed on a reduced sample size (N = 11).

Chi-squared test was used to compare variables between responders and non-responders. Tolerability was assessed considering the rate and severity of reported side effects, as well as rates and possible causes of drop-out.

The level of significance was set at 0.05. All statistical analyses were performed using the Statistical Package for the Social Sciences for Windows software version 27.1.0 (SPSS Inc., Chicago, Illinois, USA).

## 3 Results

### 3.1 Sample description

Fifteen patients were recruited from January 2021 to April 2024. One patient did not complete psychometric assessments (for personal reasons) and one dropped out after one week due to worsening depressive symptoms unrelated to the stimulation. The final sample consisted of 13 subjects (males: 69.2%; females: 30.8%, mean age: 43.1 ± 10.2 years). Table 1 shows social, demographic, and clinical variables of the final sample. According to rTMS parameters, the average motor threshold was 43.5 and the stimulation protocol was administered as planned in all patients.

**Table 1:** Demographic and clinical variables of the total sample.

Variables	Total sample (N = 13)	Responders (N = 4)	Non responders (N = 9)
**Gender (m;f)**	9 (69.2%); 4(30.8%)	2 (50%); 2(50%)	7(77.8%); 2(22.2%)
**Age**	43.08 ± 10.98	50.75 ± 9.43	39.67 ± 10.24
**Duration of illness (years)**	24.00 ± 10.24	32.75 ± 13.59	20.11 ± 5.73
**Duration of untreated illness (DUI) (years)**	5.69 ± 6.17	7.75 ± 6.18	4.78 ± 6.30
**Age at onset**	18.77 ± 8.08	18.00 ± 4.32	19.11 ± 9.51
**Family History (n/y)**	5 (31.5%); 8 (61.5%)	**4 (100%); 0 (0%)**	**1 (11.1%); 8 (88.9%)**
**Psychiatric comorbidities (n/y)**	4 (30.8%); 9(69.2%)	2 (50%); 2 (50%)	2(22.2%); 7(77.8%)
**Medical comorbidities (n/y)**	8 (61.5%); 5(38.5%)	1 (25%); 3 (75%)	7(77.8%); 2(22.2%)
**Stressors at OCD onset (n/y)**	15 (0%); 0(100%)	0 (0%); 0 (0%)	0 (0%); 0 (0%)
**Current psychological therapy (n/y)**	11 (84.6%); 2 (15.4%)	0 (0%); 4 (100%)	7 (77.8%); 2 (22.2%)
**Lifetime suicidal ideation (n/y)**	8 (61.5%); 5 (38.5%)	**4 (100%); 0 (0%)**	**4 (44.4%); 5 (55.6%)**
**Current suicidal ideation (n/y)**	12 (92.3%); 1 (7.7%)	4 (100%); 0 (0%)	8 (88.9%); 1 (11.1%)
**Number of hospitalizations**	10 (76.9%); 3 (23.1%)	3 (75%); 1 (25%)	7 (77.8%); 2 (22.2%)
**HAM-D T0**	13.23 ± 5.84	14.75 ± 5.73	12.56 ± 6.10
**HAM-A T0**	13.85 ± 7.45	19.00 ± 10.06	11.56 ± 5.12
**SDS T0**	22.69 ± 6.18	23.50 ± 5.80	22.30 ± 6.65
**CGI-S**	4.77 ± 0.83	4.50 ± 1.00	4.89 ± 0.78
**Y-BOCS T0**	23.38 ± 8.97	27.25 ± 11.52	21.67 ± 7.66

**Legend.** Bold represents a statistical significance in the comparison between responders and non-responders. CGI-S: Clinical Global Impression-Severity Scale; HAM-A: Hamilton Anxiety Rating Scale; HAM-D: Hamilton Rating Scale for Depression; SDS: Sheehan Disability Scale.; Y-BOCS: Yale-Brown Obsessive compulsive Scale. Continuous variables are expressed as mean and standard deviations (in backets).

### 3.2 Efficacy measures

#### 3.2.1 Short-term effects of the treatment

At the end of the treatment, a significant reduction on the Y-BOCS was observed among participants (T0:23.4 ± 8.9 - T1:18.2 ± 7.2, F(1,12) = 9.64; p = 0.009).

Considering the other questionnaires administered, at the end of the stimulation a statistically significant reduction of depressive symptoms was observed (HAM-D, T0:13.2 ± 5.8 - T1:9.5 ± 3.6; F(1,12) = 8.35; p = 0.014). Additionally, an improvement of anxious symptoms was registered (HAM-A T0: 13.8 ± 7.4 – T1: 8.6 ± 4, F(1,12) = 11.16; p = 0.006). Overall, patients showed a general improvement of the symptomatology, as CGI-S scores reduced at the end of the treatment (T0:4.8 ± 0.8 - T1:4.3 ± 0.9; F(1,12) = 6.35; p = 0.027). Using a subjective evaluation of the impact of the disease on quality of life, a statistical improvement was also observed at the SDS (T0: 22.7 ± 6.2 – T1: 18.3 ± 5.1; F(1,12) = 10.02; p = 0.008).

#### 3.2.2 Long term effects of the treatment

**Fig. 3:**
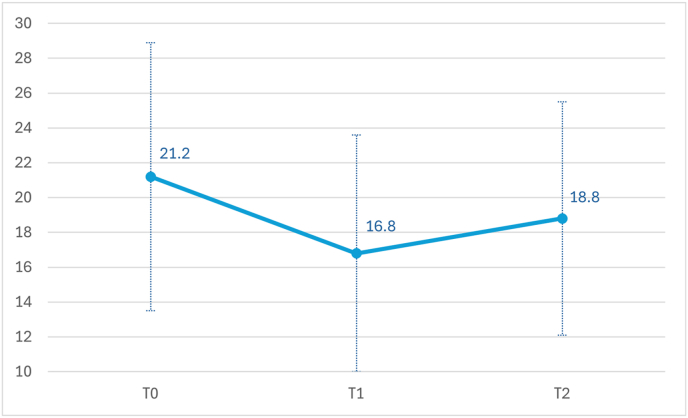
Y-BOCS scores among timepoints. Legend. Y-BOCS scores at baseline (T0), at the end of the treatment (T1) and after one month follow up (T2) in 11 patients that completed the assessment.

Comparing the three timepoints, a trend toward significant reduction on Y-BOCS scores was observed (F(1,10) = 3.94; p = 0.075). At one month follow-up, the reduction on Y-BOCS scores was still present (T2:18.2 ± 6.7), without any significant changes between T1 and T2 (p = 0.142). Fig. 3 shows the trend on the Y-BOCS at different timepoints.

With respect to the other questionnaires, HAM-D scores maintained a statistically significant improvement (F(1,10) = 5.11; p = 0.047), with pairwise comparisons indicating a significant difference between both T0 and T1 (p = 0.033) and T0 and T2 (T2: 8.7 ± 4.6; p = 0.047). Moreover, a trend toward significant reduction on HAM-A scores among the three timepoints was also observed (T2:8.1 ± 4.2, p = 0.077). Compared to baseline, anxious symptoms were significantly reduced immediately after the treatment end (p = 0.018), with a trend toward significance after one month (p = 0.077). This effect was not observed on the other psychometric assessments. Indeed, CGI-S and SDS scores did not significantly ameliorate among all timepoints (CGI-S: T2:4.3 ± 0.8; SDS T2:18.0 ± 6.5). The trend of the scores on the psychometric evaluation scales is shown in Table 2.

**Table 2:** Psychometrics assessments scores among timepoints.

	T0	T1	T2
**Y-BOCS**	21.27 (7.73)	16.82 (6.82)	18.18 (6.67)
**HAM-A**	12.45 (7.27)	7.18 (2.18)	8.09 (4.15)
**HAM-D**	12.55 (6.07)	8.82 (3.40)	8.63 (4.60)
**SDS**	21.55 (6.02)	17.09 (4.59)	18.18 (6.55)
**CGI-S**	4.55 (0.68)	4.18 (0.98)	4.27 (0.78)

**Legend.** Sample N = 11. Variables are expressed as mean and standard deviation (in brackets).

### 3.3 Responders vs. non responders

Non responders constituted most of the sample (non-responders: 69.2 (N = 9) vs. responders: 30.8% (N = 4); p = n.s.). Table 1 shows the difference between these subgroups with respect to sociodemographic and clinical variables. Regarding the statistical homogeneity of the participants, we found no significant differences in terms of age, gender, age at onset, and DUI. Compared to responders, non-responders showed some worse clinical variables. The non-responders group showed a higher rate of patients with a positive psychiatric familiarity (88.9%) and a higher rate of patients with lifetime suicidal ideation (55.6%). Statistical significance (p = 0.02) was reported among participants in the frequency of psychiatric familiarity, and a trend toward significance (p = 0.057) was reported among participants in the frequency of lifetime suicidal ideation.

### 3.4 Safety and tolerability

Mild and transient headache during stimulation was the only side effect reported by one subject (N = 1). As mentioned above, one patient dropped out from the study. This happened before the 6th session as the patient manifested a worsening of depressive symptoms that occurred after the stimulation was suspended for the weekend, during which the patient observed depressed mood, lethargy, and hypobulia, and these symptoms worsened compared to the previous weekend. The patient, worried that these symptoms were related to the rTMS, preferred to discontinue the treatment. According to our clinical judgment, no significant changes in depressive symptoms occurred (HAM-D at baseline: 8, at the time of drop out HAM-D:10) and no correlation with rTMS could be established. We hypothesize that the patient felt generally more tired after one week of daily rTMS sessions, performed in the early morning, as the patient was used to waking up and starting the day later.

## 4 Discussion

In this open-label interventional trial, we evaluated the efficacy of rTMS as an add-on treatment in patients with TR-OCD. Our results evidenced that rTMS targeting the left OFC may be beneficial for approximately one-third of patients, as indicated by a significant reduction in the Y-BOCS scores, as well as improvements in anxiety and depressive symptoms and overall global functioning. Notably, our results also suggest that the decrease of clinical symptoms is maintained at one-month after the treatment. Additionally, rTMS treatment appears to be well tolerated. Therefore, our findings support the use of rTMS targeting the left OFC as a valid treatment option in patients with TR-OCD.

As mentioned in the introduction section, limited investigations have evaluated the efficacy of rTMS targeting the OFC. Our findings are in line with previous studies, which observed a significant reduction of obsessive-compulsive symptoms in TR-OCD patients following rTMS treatment targeting this brain area (Ruffini et al., 2009), (Nauczyciel et al., 2014), (Khedr et al., 2022), (Kumar et al., 2018). In our paper, symptom improvement persisted at one-month follow-up. Literature data remains controversial on this topic, with two studies showing a maintained effect (Khedr et al., 2022), (Kumar et al., 2018), although one other reported a loss of efficacy one-month after the end of the acute treatment (Nauczyciel et al., 2014). The paper from Ruffini and colleagues showed that a beneficial effect persisted 10 weeks after the end of the rTMS sessions, but was lost at a longer 12-week follow-up (Ruffini et al., 2009). These findings suggest that further investigations with longer follow-up are needed to assess the actual duration of treatment efficacy and to determine whether maintenance sessions can contribute to a more sustained symptom relief.

In contrast with our findings, a crossover study by Zıblak and colleagues (Ziblak et al., 2021) failed to detect any significant differences between active rTMS and placebo, suggesting that rTMS may not have a discernible effect in this context. The discrepancies in these findings may be attributed to the heterogeneity of OCD symptoms and dimensions, as well as the lack of statistical power of these studies, highlighting the need for further investigation to clarify the efficacy and optimal parameters of rTMS for OCD treatment. It is noteworthy that the studies in question employed different coils. Nauczyciel et al. (2014) used a double-cone coil, whereas Zıblak et al. (Ziblak et al., 2021), Ruffini et al. (2009), Khedr et al. (2022), and Kumar et al. (2018) used an 8-shaped coil. Research has shown that the double-cone coil provides deeper stimulation compared to the 8-shaped coil (De Deng et al., 2013), (Roth et al., 2007), which may contribute to its potential efficacy (Ziblak et al., 2021). However, it is also important to acknowledge that previous studies, including our own, have used 8-shaped coil and still achieved positive results. The H-coil, designed to target primarily the medial prefrontal cortex and the anterior cingulate cortex, is the only FDA-cleared for OCD (Harmelech et al., 2021), but this coil cannot be used to target the OFC. This suggests that the optimal rTMS protocol for OCD treatment remains an open question, and further research is needed to determine the most effective approach.

Despite the growing body of research on TMS protocols, there is a limited number of studies specifically targeting the OFC. This scarcity may be attributed to the limited evidence supporting its efficacy. In contrast, most studies have focused on applying rTMS over the bilateral SMA or the DLPFC (Liang et al., 2021), (Mantovani et al., 2010), (Gomes et al., 2012), (Lusicic et al., 2018). However, previous research has consistently demonstrated bilateral or unilateral OFC hyperactivation in OCD patients (Rapinesi et al., 2019). Furthermore, studies have shown that reducing OFC hyperactivation is associated with a reduction in OCD symptoms, achieved through various treatments including SSRI (Saxena et al., 1999), cognitive-behavioral therapy (Cocchi et al., 2018), or rTMS (Nauczyciel et al., 2014), (Rapinesi et al., 2019). Our findings are consistent with these results, providing further evidence that the OFC plays a significant role in the pathophysiology of OCD and may represents a potential neuroanatomical target for rTMS treatment.

Our study reports a response rate (i.e., ≥35% reduction at the Y-BOCS) of approximately 30% after rTMS treatment. This finding is consistent with the results of a recent international survey, which found that the expected response rate for patients with OCD undergoing various TMS protocols ranged from 25% to 50% (Brakoulias et al., 2021). Intriguingly, a recent meta-analysis by Pellegrini et al. (2022) has shed new light on the effectiveness of rTMS treatment. The study found that patients with lower levels of SSRI-resistance (i.e., less than two SSRI failed trial) may benefit more from rTMS, suggesting that it would be most effective when implemented earlier in the treatment process, possibly alongside SSRI and cognitive-behavioral therapy (Pellegrini et al., 2022).

Our study revealed a statistically significant reduction of anxiety and depressive symptoms. It is noteworthy that three previously cited works, targeting the OFC, have examined this aspect. Ruffini et al. (2009) found a reduction of anxiety and depression symptoms, but not a significant difference between active and placebo group. Also, Zıblak et al. (Ziblak et al., 2021) reported no changes of these symptoms, likely due to a low score at baseline (HAM-D 4.89 ± 2.38). In the trial from Khedr and colleagues, conversely, active rTMS targeting the right OFC outperformed the sham group also on depressive and anxiety symptoms (Khedr et al., 2022). Other studies, adopting different targets and stimulation protocols, have shed the light on a positive effect on depressive symptoms when using rTMS for OCD treatment (Gomes et al., 2012), (Mansur et al., 2011), (Sarkhel et al., 2010). Furthermore, the above-mentioned meta-analysis by Pellegrini et al. (2022) found that individuals with higher baseline depression levels, as measured by the HAM-D scale, were more likely to experience a better response to rTMS treatment for OCD. Regarding the use of rTMS in treatment-resistant depression, the most benefit has been observed from high-frequency stimulation of the DLPFC (Fox et al., 2012), (Weigand et al., 2018). However, in cases of treatment resistance, OFC could represent a possible alternative (Fettes et al., 2017), (Feffer et al., 2018), (Tadayonnejad et al., 2022). The OFC is indeed also involved in mood regulation circuitry (Rolls, 2019), (Rolls and Grabenhorst, 2008). Dysfunction in OFC contributes to the pathophysiology of various psychiatric disorders such as depression, anxiety, and psychosis (Drevets, 2007), (Nakao et al., 2014), (Cheng et al., 2016). This appears to be linked to abnormal functioning within the basal ganglia "loop circuits," extending from the OFC to associated regions including the striatum, pallidum, thalamus, subthalamic nucleus, and midbrain dopaminergic structures (Drevets, 2007), (Gorwood, 2008), (Ahmari and Dougherty, 2015), (Wood and Ahmari, 2015). Therefore, it is plausible that the improvement in OCD symptoms observed in our study may also be associated with improvements in mood and anxiety. However, future research should investigate this relationship further. It is interesting to note that we observed a less marked improvement of the SDS compared to the Y-BOCS or other psychometric questionnaires, which could be explained by a later improvement of the overall well-being reported by participants. The trends of significance obtained might justify the use of a maintenance protocol for the long-term treatment of OCD patients, although stronger evidence supporting these claims is needed.

In the present investigation, we found that the presence of suicidal ideation and a positive family history of psychiatric disorders are common factors in patients who do not respond to rTMS treatment. This is consistent with previous studies showing that a positive history of psychiatric pathology has a negative impact on treatment outcomes in patients with OCD (Fontenelle et al., 2004), (Jakubovski et al., 2013). A family history of psychiatric disorders may also contribute to a lack of support during the patient's therapeutic journey, either due to unsupportive family members or an unwelcoming and dysfunctional family environment (Jakubovski et al., 2013). Our findings align with previous research, which has characterized poor responders to treatment as having high rates of psychiatric comorbidity, high baseline Y-BOCS scores, longer duration of illness, and a greater number of failed treatments (Khedr et al., 2022), (Kumar et al., 2018). This suggests that greater severity of illness may be a barrier to successful rTMS treatment. Furthermore, it can be hypothesized that comorbid depression may lead to decreased motivation to undergo treatment and avoidance behaviors (Abramowitz et al., 2000). These findings highlight the importance of considering these factors when developing treatment plans for patients with OCD. These results may also help in selecting, during the clinical evaluation, a subgroup of patients, which are more likely to benefit from rTMS treatment. It is noteworthy that the present study was conducted in a real-world clinical setting, reflecting the complex and multifaceted nature of OCD in everyday practice, where comorbidity with OCD is a common occurrence (Angst et al., 2004).

Finally, it is important to highlight the tolerability of this procedure. In our study, there was only one case of mild transient headache; this type of headache is indeed the most frequent adverse event reported in rTMS patients (Grassi et al., 2023). rTMS is a non-invasive and well-tolerated neuromodulation technique; the primary adverse effect during treatment is the potential risk of inducing seizures. However, in patients without specific risk factors, the risk is very low, fewer than 1 seizure per 60.000 sessions (Lerner et al., 2019). Patients at higher risk include those with congenital epilepsy, brain injuries, substance or alcohol abuse, or those on medications that lower the seizure threshold (Rossi et al., 2021). For this population, a careful assessment of risks and benefits is necessary before adopting this treatment strategy. In our study, all potentially at-risk patients were excluded. However, if a seizure is induced by rTMS, it occurs only during stimulation; this also applies to patients with epilepsy, who do not have an increased risk of seizures in the hours or days following treatment (Rossi et al., 2021). In a meta-analytic study comparing different target areas, rTMS applied over the OFC was the most tolerated, followed by rTMS applied over the DLPFC (Liang et al., 2021). The good tolerability of the treatment in our study is also indicated by the low dropout rate, with only one case of dropout due to worsening depressive symptoms unrelated to rTMS.

## 5 Limitations

The following limitations should be considered for a correct interpretation of the results. Firstly, the open-label design, which lacks blinding, may introduce bias due to participants' knowledge of their assigned treatment. This could influence their subjective reports of symptom improvement and potentially compromise the validity of the findings. Furthermore, the absence of a control group makes it difficult to rule out the placebo effect, which may have impacted the validity of the results. Additionally, the small sample size may have reduced the study's statistical power, making it less effective in detecting significant differences, possibly underreporting side effects and impacting the replicability of the results. In the present study, we observed a trend over significance of some outcomes measure (e.g., the Y-BOCS and the HAM-A over the one-month follow-up) which could be differently affected by a larger sample size or a longer duration of the observation. Furthermore, pairing rTMS with functional neuroimaging (e.g., fMRI or PET scans) might have led to more accurate targeting of the left OFC and a neuroimaging-guided protocol could have improved the precision and reproducibility of the treatment. Pairing functional neuroimaging with rTMS stimulation offers a step further to improve the precision and therefore efficacy of rTMS treatment.

## 6 Conclusion

Mostly used as a non-pharmacological treatment for depressive episodes, rTMS has been also employed in various psychiatric conditions such as OCD, with evidence of its efficacy and tolerability in multiple studies. Target areas, duration of stimulation, intensity, frequency of stimulation, and clinical variables are currently being analyzed in order to assess the most adequate protocol. In this study we assessed the efficacy and tolerability of 1 Hz rTMS targeting the left OFC on a sample of TR-OCD patients, with one third of the sample showing response to the treatment. Efficacy at the end of the treatment was confirmed employing statistical analysis, and maintenance of the effects of the stimulation was observed after a month follow-up. In the future larger, blinded, and controlled studies are needed to further validate present findings. Furthermore, the outcomes emerged in the present work could be improved in future work through several approaches: i) possibly increasing the stimulation intensity, ii) increasing the number of pulses, iii) increasing the number of sessions or the number of sessions per day to accelerate the course of treatment, and iv) implementing with maintenance rTMS sessions to sustain the effect over a longer follow-up.

## Disclosures

Prof. Dell'Osso has received Grant/Research Support from LivaNova, Inc., Angelini and Lundbeck and Lecture Honoraria from Angelini, Janssen, Otzuka and Lundbeck. Dr Vismara, Dr Torriero, Dr La Monica, Dr Benatti, Dr Larini, Dr Bucca, Dr. Bosi, and Dr Girone have no conflict of interest to report.

## Funding source

The present article has not been funded.
